# A large-scale prediction model to predict large for gestational age infants conceived by IVF/ICSI

**DOI:** 10.3389/fendo.2026.1842531

**Published:** 2026-07-14

**Authors:** Xiuyun Li, Aijuan Zhang, Gang Bai, Yue Liu, Wenlan Xing, Yan Li

**Affiliations:** 1 Infection and Microbiology Research Laboratory for Women and Children, Shandong Provincial Maternal and Child Health Care Hospital Affiliated to Qingdao University, Jinan, China; 2 Obstetrics and Gynecology Department, Shandong Provincial Maternal and Child Health Care Hospital Affiliated to Qingdao University, Jinan, China; 3 State Key Laboratory of Reproductive Medicine and Offspring Health, Center for Reproductive Medicine, Institute of Women, Children and Reproductive Health, Shandong University, Jinan, China; 4 Clinical Research Center for Child Health and Disorders, Shandong Provincial Maternal and Child Health Care Hospital Affiliated to Qingdao University, Jinan, China

**Keywords:** clinical prediction model, *in vitro* fertilization, intracytoplasmic sperm injection, large for gestational age, XGBoost

## Abstract

**Objective:**

To develop and internally validate a machine learning model for predicting the risk of large-for-gestational-age (LGA) birth following IVF/ICSI and to identify important parental and treatment-related predictors.

**Methods:**

A total of 17, 741 singleton live births resulting from IVF/ICSI, categorized as appropriate for gestational age (AGA) or LGA. Data on birth outcomes, parental characteristics, and treatment-related variables were collected. The dataset was randomly divided into training and testing sets (7:3). An XGBoost model was developed and optimized using the Optuna framework and Tree-structured Parzen Estimator algorithm. Model performance was evaluated using the area under the receiver operating characteristic curve (AUC), calibration analysis, Brier score, and classification metrics. Logistic regression was used as a conventional baseline model. Model interpretability was assessed using SHAP analysis.

**Results:**

The XGBoost model achieved an AUC of 0.7003 in the internal hold-out test set, compared with 0.6445 for logistic regression. Calibration analysis showed a lower Brier score for XGBoost than for logistic regression (0.2040 vs. 0.2295). SHAP analysis identified embryo transfer strategy, maternal anthropometric and metabolic characteristics and several paternal characteristics as important predictors in the model.

**Conclusions:**

The machine learning model demonstrated moderate discriminative ability for predicting LGA risk among IVF/ICSI-conceived singleton births in internal validation. The findings highlight the dominant association of embryo cryopreservation strategies, maternal and paternal factors with fetal overgrowth. While the model shows acceptable discriminatory ability, its clinical utility requires future prospective external validation.

## Graphical Abstract

**Figure:**
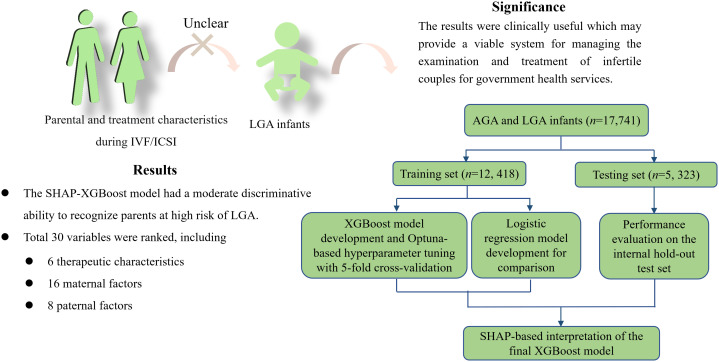

## Introduction

The widespread application of optimized ovarian stimulation protocols and cryopreservation techniques has facilitated the global expansion of assisted reproductive technology (ART) for infertility treatment. Improvements in the success rates of popular ART treatments such as *in vitro* fertilization (IVF) and intracytoplasmic sperm injection (ICSI) have brought new hope to infertile adults. It is estimated that ART has contributed to more than 10 million live births worldwide, of which more than 1% have occurred in China (1, 2).

Although the majority of children conceived by IVF/ICSI are healthy, these techniques are associated with increased risks of adverse obstetric and perinatal outcomes compared to natural conception. These adverse outcomes include higher perinatal mortality, preterm birth, low birth weight (LBW), and large for gestational age (LGA) (3–5). LGA, defined as birthweight above the 90th percentile, poses risks to both the mother and infant during delivery and to the growth and development of the child after delivery (6). LGA is associated with significant neonatal morbidity and mortality due to the increased difficulty of the passage of the infant through the bony maternal pelvis, which can lead to shoulder dystocia and associated birth asphyxia (7). Additionally, LGA is linked to long-term health issues, such as obesity, asthma, type 2 diabetes, metabolic syndrome, and cardiovascular disease (8, 9). Mothers delivering LGA infants are vulnerable to prolonged childbirth and increased complications, such as postpartum hemorrhage, soft tissue injury, perineal tears, and emergency cesarean section (7, 10, 11). Given these adverse outcomes, it is essential to understand the factors contributing to LGA in infants conceived by IVF/ICSI.

An extensive body of work has identified a number of maternal and paternal factors that influence the rates of LGA outcomes in natural pregnancy, such as parental body mass index (BMI), maternal gestational diabetes, maternal obesity or excessive weight gain during pregnancy and erythroblastosis fetalis (12). However, our comprehensive search for studies of LGA infants conceived by IVF/ICSI technology without language restrictions in two databases revealed that research on IVF/ICSI-conceived infants and the baseline characteristics of their parents has largely focused on small for gestational age (SGA) infants rather than LGA infants (13–16). International evidence suggests that the rates of LGA outcomes are higher for infants from frozen embryos than those from fresh embryos (5, 17, 18), and several studies have noted that the BMI of the father or mother may be associated with LGA outcomes in infants conceived by IVF/ICSI (19, 20). Crucially, emerging mechanistic evidence indicates that paternal factors play a non−negligible role specifically in IVF/ICSI. Sperm epigenetics (DNA methylation, non−coding RNAs) can be altered by paternal obesity or metabolic syndrome. And the ICSI procedure bypasses natural sperm selection barriers (e.g., zona pellucida binding and sperm competition), allowing potentially abnormal molecular signals to be transmitted directly to the embryo (21, 22). Thus, understanding the relative contributions of intrinsic parental characteristics would improve IVF/ICSI outcomes and further advance clinical practice.

Currently, there is no standardized tool for identifying couples at high risk of having LGA infants through IVF/ICSI. Moreover, most studies of abnormal newborn weight outcomes have investigated basic maternal characteristics, and there is a lack of research on important paternal physical indicators, particularly paternal metabolic profiles, reproductive endocrinology, and detailed semen parameters. There is growing evidence that correlations of male reproductive factors with offspring birth outcomes cannot be ignored. In addition, LGA results from complex, non−linear interactions among multiple parental and treatment variables. Traditional regression models have limited ability to capture such interactions. The XGBoost algorithm, combined with SHAP analysis, has been successfully applied in perinatal medicine and provides both high predictive accuracy and model interpretability, making it suitable for constructing a clinically useful prediction model (23). Therefore, the aim of this study was to identify paternal and maternal risk factors for LGA infants conceived by IVF/ICSI and to construct a relevant predictive model to provide an auxiliary and practical reference for clinical use.

## Materials and methods

### Data sources

This study was a single-center retrospective cohort study conducted at the Center for Reproductive Medicine of Shandong University, which was approved by the ethical committee of the Center for Reproductive Medicine of Shandong University (2022 IRB number 101). The birth and follow-up information of infants conceived by IVF/ICSI, the characteristics of their parents before and after embryo transfer, and treatment characteristics were collected. Based on egg retrieval dates, case data were collected from January 1st, 2014 to January 1st, 2018. All data were extracted from the electronic medical information system, eliminating the need for informed consent from participants. All examined infants were conceived through IVF/ICSI using autologous gametes.

According to the growth reference standards and curves of the birthweight, length, and head circumference of neonates of different gestational ages in China, the infants conceived by IVF/ICSI were classified as SGA, AGA or LGA (24). This study only analyzed data from AGA and LGA single live births conceived by IVF/ICSI for whom complete birth information was available. The final cohort included 17, 741 singleton births, including 5, 521 LGA events. The number of outcome events substantially exceeded commonly recommended events-per-variable criteria for prediction model development, supporting model stability and reducing the risk of overfitting (25). The specific inclusion criteria were singleton live birth AGA and LGA infants conceived by IVF/ICSI with complete birth information. The specific exclusion criterion was incomplete parental indicators.

Maternal and paternal demographic, clinical, laboratory, and treatment-related variables available in the electronic medical record system were considered as candidate predictors. Smoking-related and diabetes-related variables available in the database were included in model development. However, information regarding gestational diabetes mellitus and gestational weight gain was not available in the database and therefore could not be evaluated in the present study.

### Definitions

Gestational age at delivery in IVF/ICSI pregnancies was calculated by adding 14 days to the number of days between the date of oocyte retrieval and the date of delivery. AGA was defined as birthweight between the 10th and 90th percentiles of the average birthweight for the same gestational age. LGA was defined as birthweight above the 90th percentile of the gestational age-specific birthweight distribution (24).

### Statistical analysis

**Figure 1:**
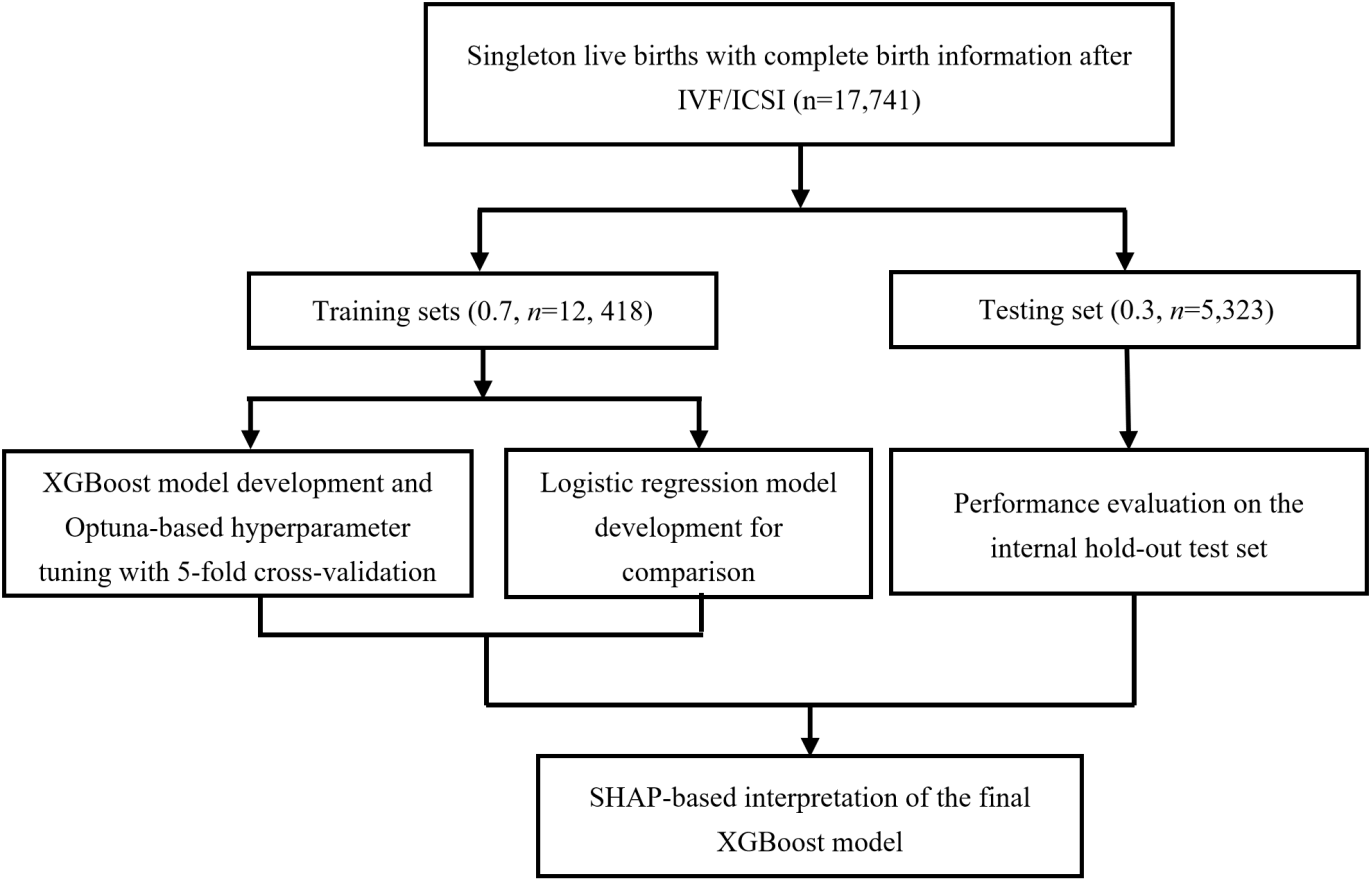
Flowchart of this study.

Initially, we partitioned the entire dataset into a training set and an independent test set using a 7:3 stratified sampling ratio (**Figure 1**). The test set was held out and completely isolated during the model training and optimization phases, reserved solely for the final performance evaluation. A ratio of 7:3 is common for dividing data into training and testing sets for machine learning. This ratio effectively balances the amount of training and testing data to ensure the stability and generalizability of the model and has been used in many high-impact studies (26–28). On the training set, we chose the XGBoost algorithm, an efficient gradient boosting model. We employed the Optuna framework combined with the Tree-structured Parzen Estimator (TPE) algorithm to automate the optimization of the model’s key hyperparameters. The objective was to maximize the AUC under 5-fold stratified cross-validation performed within the training set. The optimized parameters and their search ranges included: maximum tree depth (max_depth: 3-10), learning rate (learning_rate: 0.005-0.2), number of estimators (n_estimators: 200-1200), minimum child weight (min_child_weight: 1-20), subsample ratio of training instances (subsample: 0.3-1.0), subsample ratio of columns for each tree (colsample_bytree: 0.2-1.0), minimum loss reduction required for a split (gamma: 0.0-5.0), L1 regularization (reg_alpha: 1e-8-10.0), and L2 regularization (reg_lambda: 1e-3-100.0). Concurrently, we effectively handled the class imbalance problem by setting the scale_pos_weight parameter. After determining the optimal hyperparameters, the final model was trained on the entire training set using these parameters. Finally, model performance was evaluated on the independent test set. Discriminative ability was assessed using the area under the receiver operating characteristic curve (AUC), precision, recall, F1-score, and confusion matrix. To assess calibration, calibration curves and Brier scores were additionally calculated. Logistic regression was implemented as a conventional baseline model for comparison using the same training and testing datasets. To gain a deeper understanding of the model’s internal decision-making mechanism, we introduced the game theory-based SHapley Additive exPlanations (SHAP) method. To avoid potential data leakage, variables that could only be determined after pregnancy outcome assessment, including preterm birth, were excluded from model development and validation. By analyzing global feature importance and dependency plots for key features, we achieved interpretability for the model’s prediction results. The entire analysis was conducted in the Python environment, and a global random seed (123) was set to ensure the reproducibility of the results.

## Results

Characteristics of the infant sample: A total of 17, 741 singleton live births resulting from IVF/ICSI were included in the analysis. Male infants comprised 53.2% (n=9, 437) of this sample. The dataset included 12, 220 AGA infants and 5, 521 LGA infants. The median paternal age was 30 years, and the median maternal age was 29. The training set comprised 12, 418 infants, and the testing set included 5, 323 infants. The prevalence of LGA was 31.12% in the training set and 31.13% in the testing set.

Demographic and clinical characteristics of the AGA and LGA groups are summarized in **Supplementary Table 1**. Continuous variables are presented as median with interquartile range (IQR). Compared with AGA infants, mothers of LGA infants had higher body weight (59.0 [53.1–65.5] vs. 62.4 [56.0–70.0] kg, p < 0.001) and higher body mass index (22.61 [20.55–25.15] vs. 23.73 [21.48–26.50] kg/m², p < 0.001). Paternal body weight and body mass index were also significantly higher in the LGA group (both p < 0.001). Detailed comparisons for all variables, including statistical tests and exact p−values, are provided in **Supplementary Table 1**.

### Model performance

**Figure 2:**
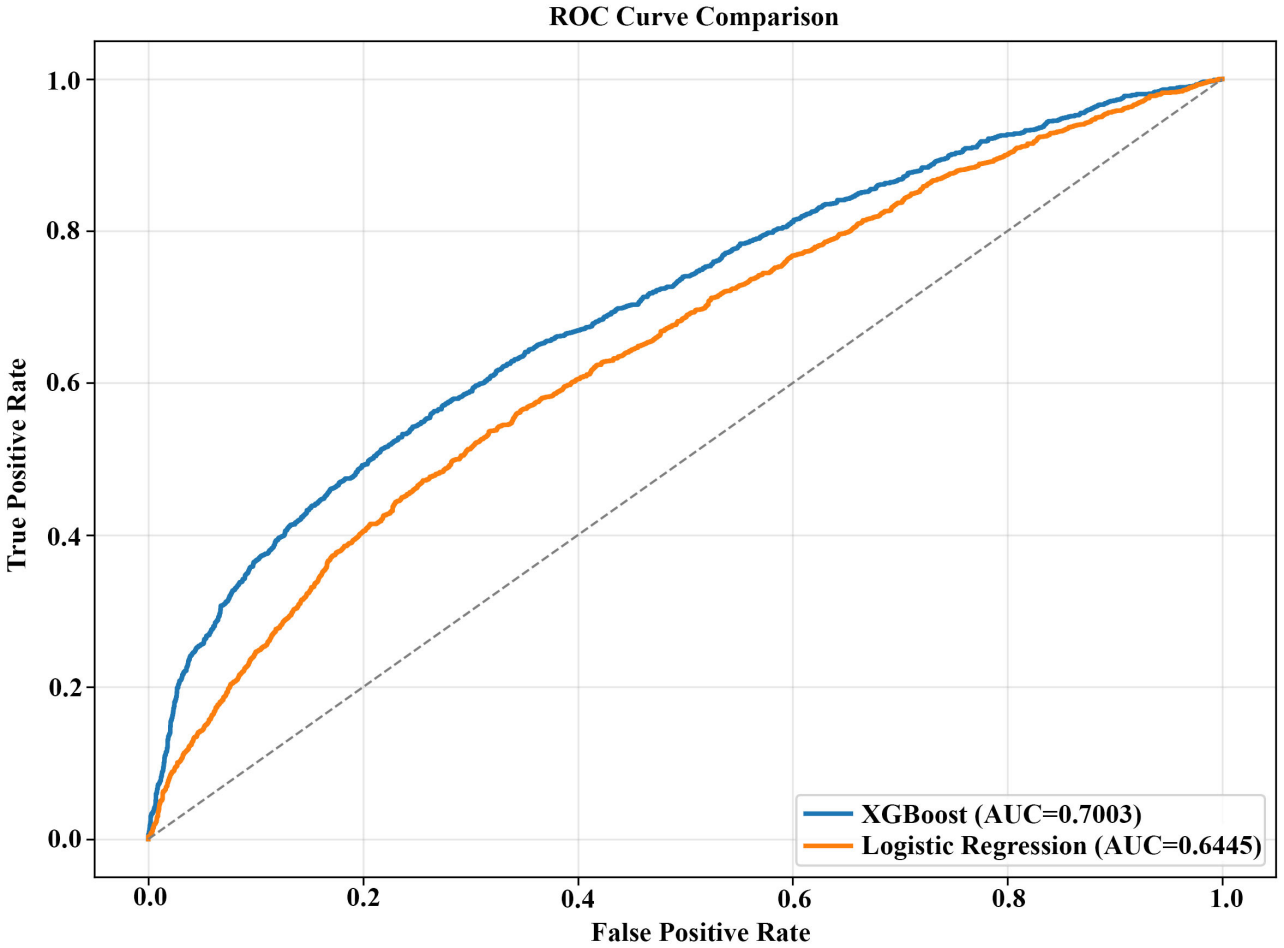
ROC curve of XGBoost and logistic regression.

**Figure 3:**
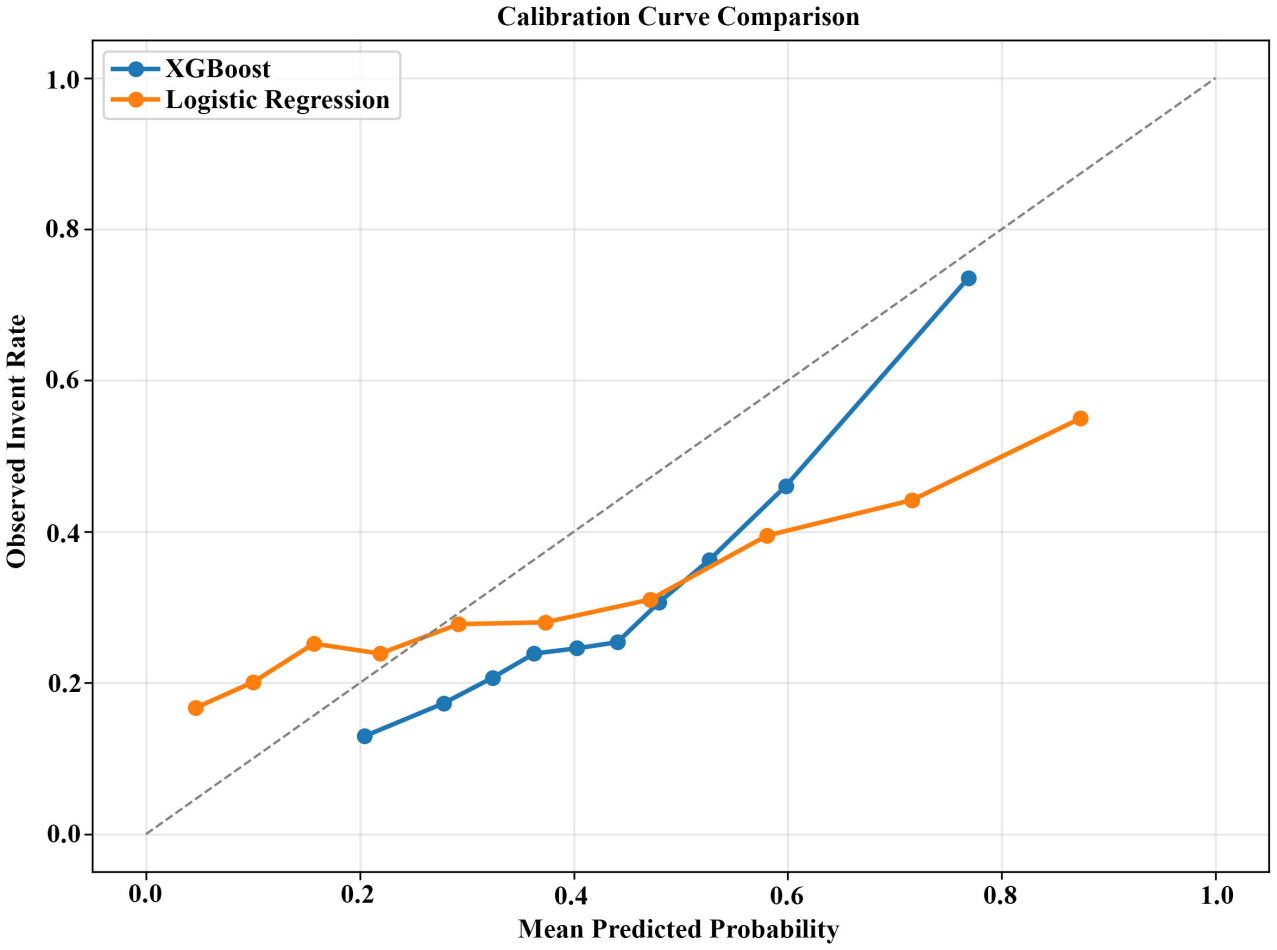
Calibration curve comparison.

The XGBoost model demonstrated moderate discriminative ability for predicting LGA among IVF/ICSI-conceived singleton births. In the internal hold-out test set, the model achieved an AUC of 0.7003. Under the same outcome definition and train-test split, logistic regression achieved an AUC of 0.6445, indicating inferior discriminative performance compared with XGBoost. The receiver operating characteristic (ROC) curves of both models are shown in **Figure 2**. Calibration analysis demonstrated better agreement between predicted and observed probabilities for the XGBoost model than for logistic regression (**Figure 3**). Consistently, the XGBoost model showed a lower Brier score than logistic regression (0.2040 vs. 0.2295), suggesting improved overall probabilistic prediction performance. The classification report for the LGA group showed a precision of 0.52, a recall of 0.53, and an F1-score of 0.53, reflecting a balanced ability to distinguish between LGA and AGA cases.

### SHAP analysis

**Figure 4:**
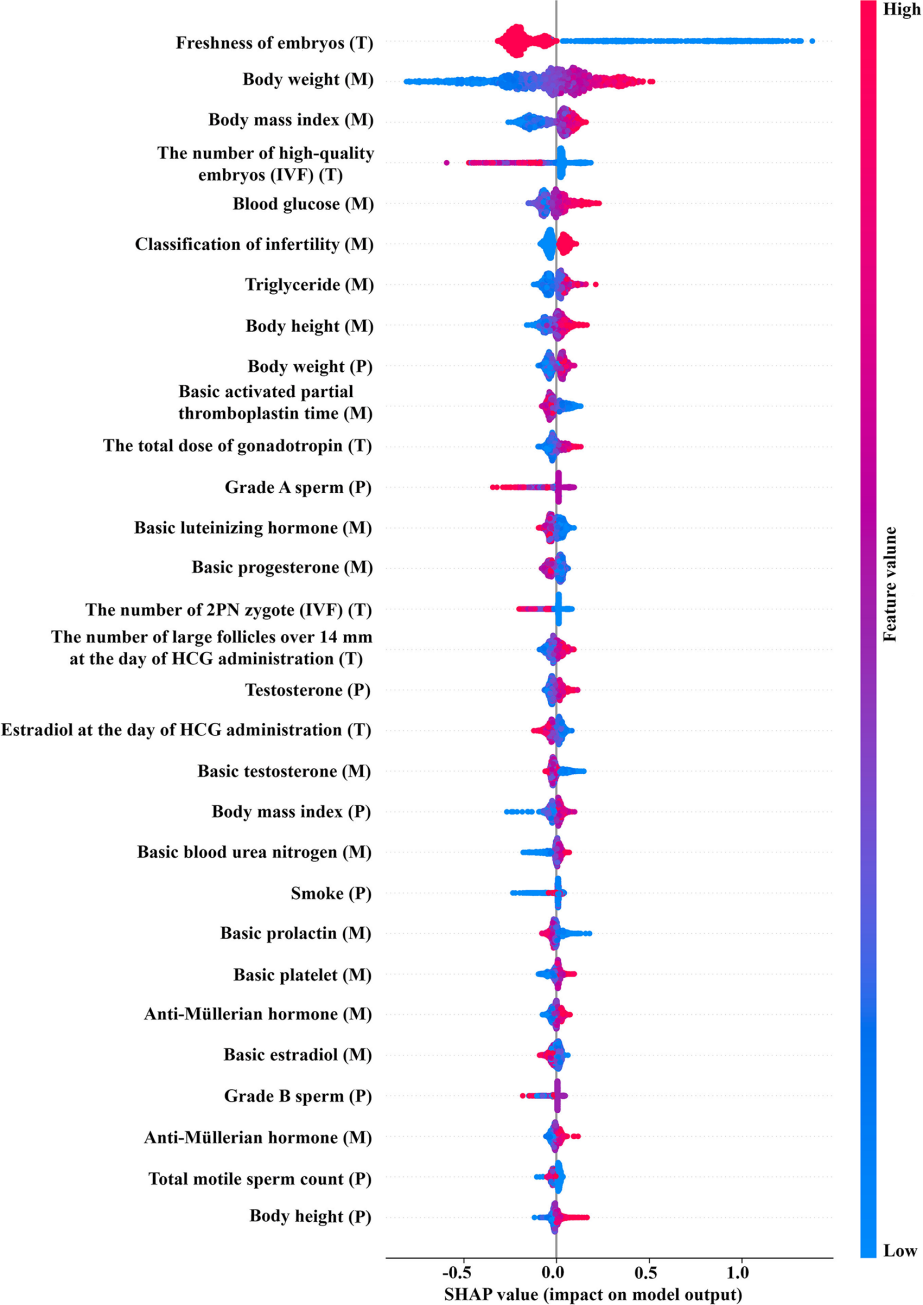
SHAP summary plot for features in the original XGBoost model. T, therapeutic characteristics; M, maternal factors; P, paternal factors.

**Figure 5:**
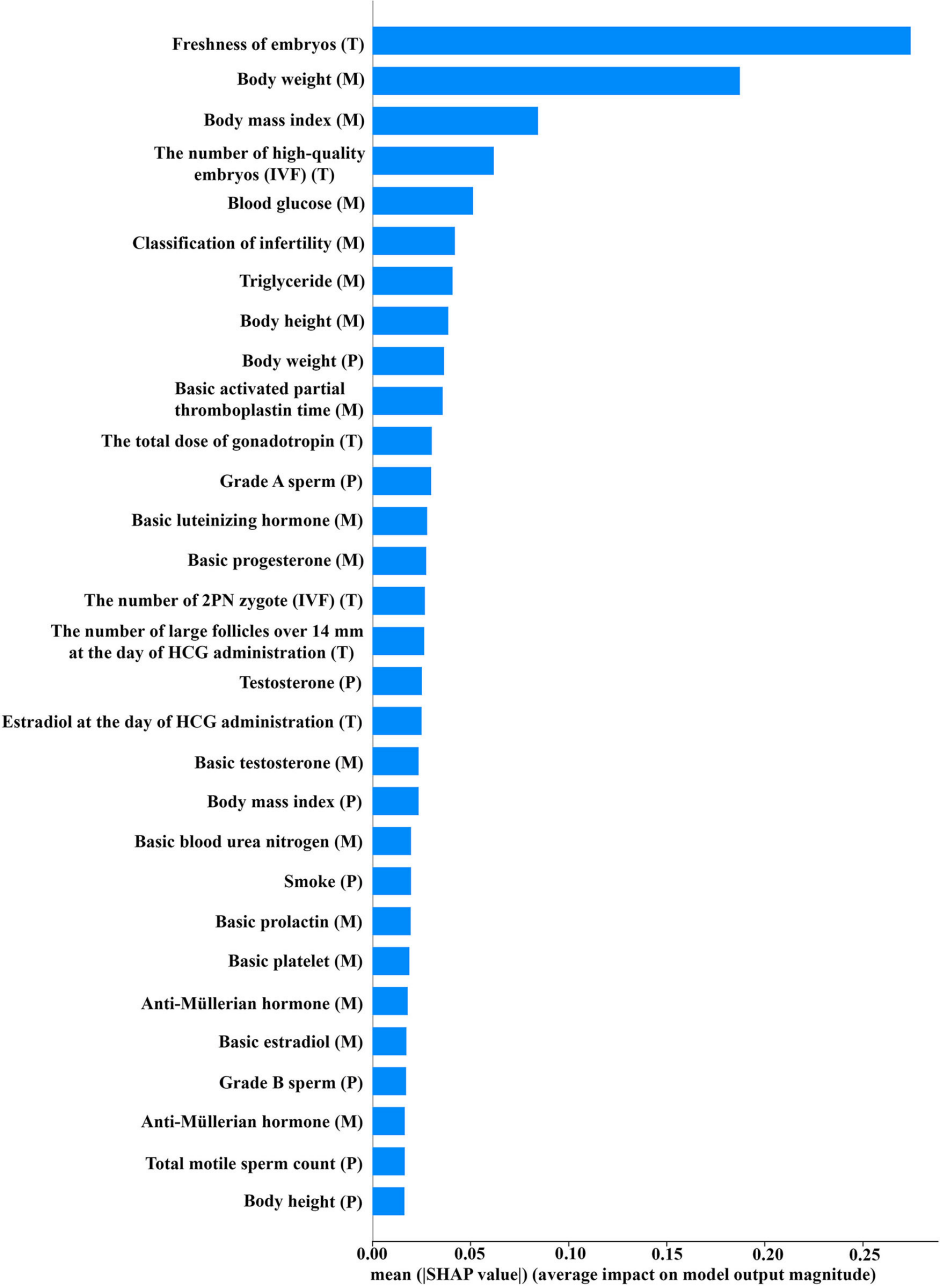
SHAP bar plot for features in the original XGBoost model. T, therapeutic characteristics; M, maternal factors; P, paternal factors.

SHAP analysis quantified the contribution of each feature to the model’s predictions and provided clinical interpretability. **Figures 4** and **5** displayed the SHAP summary plot and bar plot, respectively. In these plots, the SHAP value indicates the direction and magnitude of a feature’s impact on the predicted LGA risk: positive SHAP values increase the predicted probability of LGA, whereas negative SHAP values decrease it.

Embryo freshness (frozen vs. fresh) showed the largest positive SHAP contribution, meaning that frozen embryo transfer was the strongest predictor of LGA in the model. Maternal anthropometric and metabolic factors, including maternal body weight, body mass index (BMI), triglycerides, and glucose-related indicators, also exhibited consistently positive SHAP values. Higher values of these features were associated with an increased predicted probability of LGA. In contrast, higher embryo quality and a greater number of two-pronuclei (2PN) zygotes displayed negative SHAP values, indicating that these features contributed to a lower predicted LGA probability. Among paternal factors, paternal body weight and testosterone level yielded positive SHAP values. From a clinical perspective, SHAP analysis helps clinicians understand not only which factors are important for model prediction but also whether a given factor increases or decreases the model’s estimated likelihood of LGA in IVF/ICSI pregnancies.

## Discussion

To our knowledge, the present study is among the few to comprehensively analyze paternal, maternal, and treatment-related predictive factors for LGA in IVF/ICSI conceptions using a machine learning approach. By leveraging a large real-world dataset of 17, 741 live births, we developed a machine learning model that achieved an AUC of 0.7003 in internal validation, demonstrating moderate discriminative ability for estimating LGA risk among IVF/ICSI-conceived singleton births. Our SHAP analysis revealed that the risk of LGA is multifactorial, driven primarily by embryo freshness (frozen vs. fresh) and maternal anthropometric and metabolic characteristics (weight, BMI, glucose, triglycerides). Uniquely, our study also identified paternal body weight as a relevant predictor, highlighting the potential contribution of paternal factors to offspring growth, albeit to a lesser extent than maternal factors.

### Therapeutic characteristics

Frozen embryo transfer (FET) is associated with higher risk of LGA compared to fresh embryo transfer, consistent with extensive international evidence (29, 30). Several mechanisms may explain this phenomenon. First, the cryopreservation process and the composition of cryoprotectants might induce epigenetic alterations in the embryo, specifically affecting imprinted genes involved in growth regulation (31). Second, the freezing and thawing process may act as a selection pressure, where only the most robust embryos with the highest growth potential survive and implant. This finding is further demonstrated by other embryo-related parameters. Notably, higher embryo quality and a greater number of two pronuclei (2PN) zygotes are correlated with a lower proportion of LGA, suggesting that intrinsic embryonic vigor may be linked to more regulated postnatal growth. Indicators of ovarian response also showed distinct associations with LGA risk, collectively highlighting the influence of the stimulation environment. Specifically, a greater number of large follicles on human chorionic gonadotropin (HCG) day correlated positively with LGA, whereas higher estrogen levels on HCG day showed a negative correlation. Furthermore, a higher total dose of gonadotropins was associated with an increased probability of LGA, potentially reflecting longer stimulation duration and greater hormonal exposure, though the precise underlying mechanisms remain unclear.

### Maternal factors

Maternal anthropometric and metabolic profiles were critical predictors of LGA risk. Maternal obesity creates an intrauterine environment characterized by an abundance of nutrients, which crosses the placenta and stimulates fetal insulin secretion, a potent growth factor (32). Beyond simple obesity, our model identified blood glucose and triglycerides (TG) as key predictors within the top 10 features. This suggests that maternal lipid and glucose metabolism, even within non-diabetic ranges, plays a critical role in fetal overgrowth. Specifically, elevated maternal triglycerides can be hydrolyzed by placental lipases, increasing the free fatty acid supply to the fetus and thereby driving fat accumulation and LGA (33), which highlights the importance of comprehensive metabolic monitoring. In addition, greater maternal height was associated with an increased likelihood of delivering an LGA infant, aligning with previous reports (34, 35). Furthermore, the type of infertility was also significant, with secondary infertility linked to a higher LGA risk compared to primary infertility. This likely reflects the “parity effect, “ where multiparous women (characteristic of secondary infertility) tend to have heavier babies due to physiological adaptations in the uterus and placental efficiency established during previous pregnancies (36). Ovarian function also plays a critical role, as ovarian dysfunction often disrupts the maternal metabolic and physiological homeostasis, which serves as a critical factor driving excessive fetal growth (37, 38). In our study of women conceiving through IVF/ICSI, lower basal levels of luteinizing hormone, progesterone, testosterone prolactin and estradiol correlated with a higher likelihood of LGA. Conversely, higher levels of anti-Müllerian hormone (AMH) are associated with an increased rate of LGA newborns. This aligns with the significantly higher proportion of LGA infants among women with polycystic ovary syndrome (PCOS) (39), a condition marked by elevated AMH (40). Among other significant predictors, a shortened baseline activated partial thromboplastin time (APTT) was associated with LGA, potentially reflecting a hypercoagulable state or enhanced placental perfusion in specific subgroups. Additionally, elevated baseline blood urea nitrogen and platelet levels were linked to an increased probability of LGA, though the precise mechanisms require further elucidation.

### Paternal factors

We found that paternal body weight and paternal BMI were among the top predictive features. Paternal obesity has been shown to alter sperm DNA methylation patterns and non-coding RNA content, which can influence metabolic programming and growth trajectories in the offspring. Furthermore, emerging evidence suggests that paternal weight represents a modifiable risk factor for LGA infants (41, 42). Consistent with this, our prediction model identified paternal weight as contributing to LGA risk following IVF/ICSI conception, though this contribution was substantially less pronounced than that of maternal factors. This differential impact is further supported by a recent large-scale cohort study, which demonstrated that unhealthy maternal y BMI generally exerts stronger effects on adverse birth outcomes than paternal BMI. Notably, the study revealed that within couples where the father maintains normal weight, maternal obesity demonstrates a stronger association with adverse birth outcomes than paternal obesity under comparable conditions (42). Beyond anthropometric measures, paternal hormonal profiles also appear relevant. Testosterone, the primary male sex hormone, plays crucial roles in maintaining physiological and psychological characteristics throughout life (43, 44). Our results indicate that higher paternal testosterone levels are significantly associated with increased LGA risk, suggesting a potential dose-response relationship. To date, no other studies have confirmed this association. However, research suggest that low testosterone levels contribute to obesity, which in turn further reduces testosterone levels (45–47). This appears paradoxical in relation to the positive correlation observed between male body weight and the occurrence of LGA (42). Therefore, the relationship between testosterone and LGA risk warrants further investigation to reconcile these findings. Together, these findings underscore the importance of evaluating paternal endocrine status in both clinical counseling and assisted reproduction research to comprehensively assess offspring health risks. Currently, no study has clearly demonstrated a relationship between sperm quality and LGA. However, research has indicated that paternal obesity affects sperm quality and is associated with an increased risk of LGA (48). Our study suggests that a higher proportion of Grade A sperm is associated with a lower predicted probability of LGA. In addition, semen parameters further contribute to paternal influence on LGA risk. Both higher and lower semen volumes have been associated with reduced fertility (49), and high volume may reflect active secretion due to accessory gland inflammation (50). Our nomogram proposed a positive correlation between semen volume and LGA probability in IVF/ICSI-conceived infants, possibly mediated by accessory gland overactivity. Furthermore, diminished sperm motility was associated with a higher proportion of LGA offspring following IVF/ICSI conception. While direct clinical evidence is still lacking, we propose that reduced motility may indicate deeper molecular abnormalities in sperm, including DNA damage and epigenetic alterations (51–53). These molecular defects often originate from paternal health conditions such as obesity or metabolic syndrome. Through assisted reproductive technologies like ICSI, these otherwise “suboptimal” sperm—which might have been eliminated under natural selection—are enabled to fertilize, potentially transmitting erroneous molecular information to the embryo. In addition, we incorporated smoking status as a potential influencing factor. However, due to the extreme scarcity of positive samples (17, 731:10), SHAP was unable to accurately quantify the contribution of this feature to the XGBoost model’s predictions.

### Clinical implications

The present model may provide preliminary support for risk assessment in IVF/ICSI pregnancies. However, given the retrospective single-center design and the absence of external validation, the model should be considered exploratory and requires further validation before clinical implementation. The identification of maternal metabolic characteristics and embryo transfer strategy as major predictive features suggests that these factors may be associated with fetal overgrowth in IVF/ICSI pregnancies. These findings support the potential value of comprehensive maternal metabolic assessment during assisted reproductive treatment. In addition, the observed associations between frozen embryo transfer and increased LGA risk may warrant closer clinical attention in specific patient populations. Most importantly, our study highlights the significant influence of paternal factors on the risk of LGA, which brings new implications for clinical practice. Future multicenter and prospective studies are needed to further validate these findings and to determine whether incorporating paternal variables could improve individualized risk assessment models in reproductive medicine.

### Strengths and limitations

The major strengths of this study include the relatively large sample size and the inclusion of paternal data, which are less frequently evaluated in studies of perinatal outcomes following IVF/ICSI. In addition, the application of the XGBoost algorithm combined with SHAP analysis facilitated the exploration of complex feature relationships and model interpretability. Several limitations should also be acknowledged. First, this was a retrospective single-center study, and the generalizability of the findings remains to be validated in external and prospective cohorts. Second, information regarding gestational weight gain and gestational diabetes mellitus, both of which may influence fetal growth, was unavailable in the current database and therefore could not be incorporated into model development. Although smoking-related variables were available, the extremely low prevalence of smoking in the study population limited their contribution to model prediction. Third, although paternal anthropometric and hormonal variables contributed to model prediction, the underlying biological mechanisms require further investigation. Finally, the model demonstrated moderate discriminative performance (AUC = 0.7003), indicating that additional genetic, environmental, and pregnancy-related factors not captured in the present study may also contribute to LGA risk.

## Conclusions

In conclusion, we developed an XGBoost-based model that demonstrated moderate discriminative ability for estimating the risk of LGA among singleton infants conceived through IVF/ICSI. The model identified embryo transfer strategy, maternal body weight, body mass index, triglycerides, glucose-related indicators, along with several paternal variables, as key predictors. These findings suggest that both parental characteristics and treatment-related factors may be associated with LGA risk in IVF/ICSI pregnancies. Further external validation and prospective studies are required to confirm the generalizability and potential clinical utility of the model.

## Data Availability

The raw data supporting the conclusions of this article will be made available by the authors, without undue reservation.
